# Evaluating the Bioactivity of a Novel Broad-Spectrum Antimicrobial Peptide Brevinin-1GHa from the Frog Skin Secretion of *Hylarana guentheri* and Its Analogues

**DOI:** 10.3390/toxins10100413

**Published:** 2018-10-13

**Authors:** Qi Chen, Peng Cheng, Chengbang Ma, Xinping Xi, Lei Wang, Mei Zhou, Huimin Bian, Tianbao Chen

**Affiliations:** 1 School of Pharmacy, Nanjing University of Chinese Medicine, Nanjing 210000, China; qchen10@qub.ac.uk (Q.C.); pcheng427@njucm.edu.cn (P.C.); 2 Natural Drug Discovery Group, School of Pharmacy, Queen’s University, Belfast BT9 7BL, Northern Ireland, UK; c.ma@qub.ac.uk (C.M.); x.xi@qub.ac.uk (X.X.); l.wang@qub.ac.uk (L.W.); m.zhou@qub.ac.uk (M.Z.); t.chen@qub.ac.uk (T.C.)

**Keywords:** amphibian, antimicrobial, peptides, Brevinin-1, Rana-box

## Abstract

Many antimicrobial peptides (AMPs) have been identified from the skin secretion of the frog *Hylarana guentheri* (*H.guentheri*), including Temporin, Brevinin-1, and Brevinin-2. In this study, an antimicrobial peptide named Brevinin-1GHa was identified for the first time by using ‘shotgun’ cloning. The primary structure was also confirmed through mass spectral analysis of the skin secretion purified by reversed-phase high-performance liquid chromatography (RP-HPLC). There was a Rana-box (CKISKKC) in the C-terminal of Brevinin-1GHa, which formed an intra-disulfide bridge. To detect the significance of Rana-box and reduce the hemolytic activity, we chemically synthesized Brevinin-1GHb (without Rana-box) and Brevinin-1GHc (Rana-box in central position). Brevinin-1GHa exhibited a strong and broad-spectrum antimicrobial activity against seven microorganisms, while Brevinin-1GHb only inhibited the growth of *Staphylococcus aureus* (*S. aureus*), which indicates Rana-box was necessary for the antimicrobial activity of Brevinin-1GHa. The results of Brevinin-1GHc suggested transferring Rana-box to the central position could reduce the hemolytic activity, but the antimicrobial activity also declined. Additionally, Brevinin-1GHa demonstrated the capability of permeating cell membrane and eliminating biofilm of *S. aureus*, *Escherichia coli (E. coli*), and *Candida albicans* (*C. albicans*). The discovery of this research may provide some novel insights into natural antimicrobial drug design.

## 1. Introduction

On account of the widespread use and misuse of antibiotics during the past decades, there is an alarming emergence of strains of pathogenic bacteria and fungi that are resistant to a range of formerly conventionally used effective antibiotics, which have posed a severe global threat to public health [1,2,3,4]. As a result, there is an urgent need for some entirely novel types of antimicrobial agents which has clear pharmacokinetics and toxicology [5].

A big-sized frog *Hylarana guentheri*, which is widely distributed and the dominating amphibian species in Southern China, belongs to the genus *Hylarana* within the family Ranidae [6,7]. To adapt to specific environments, the skin secretion of *H. guentheri* contain abundant types of antimicrobial peptides(AMPs), and according to the structural characteristics, most of them belong to Brevinin-1, Brevinin-2, and Temporin families [8].

The first identified Brevinin-1 peptide was isolated from the skin of Rana *brevipoda porsa* and contained 24 amino acid residues [9]. Then some researches revealed some common structural features of Brevinin-1 family, Proline is often the 14th residue in the sequence [10], and most of them have a cyclic heptapeptide (Cys^18^-(Xaa)4-Lys-Cys^24^) called Rana-box at the C-terminal, which forms a disulfide bridge [11]. Brevinin-1 peptides usually demonstrate a broad-spectrum antimicrobial activity against Gram-positive and Gram-negative bacteria and strains of pathogenic fungi [9], and some analogues can inhibit the growth of cancer cell lines [12]. Peptides in the Brevinin-1 family are linear, amphipathic and cationic. Cationic amino acids can facilitate the interaction of peptides with the bacterial membranes and the negatively charged cell membranes through electrostatic attraction [13]. In the hydrophobic membrane-mimetic environment, Brevinin-1 peptides exist as an amphipathic α-helical structure that perturbs the phospholipid bilayer to combine well with targeted membranes [14].

In the present study, a novel peptide named Brevinin-1GHa was isolated from the skin secretion of *H. guentheri*. The primary structure was obtained via ‘shotgun’ cloning and confirmed by mass spectrum (MS/MS) fragmentation sequencing. Also, Brevinin-1GHb (without Rana-box) was designed to test the significance of Rana-box for the antimicrobial activity and Brevinin-1GHc (Rana-box in the central position) was designed to reduce the hemolytic activity. Both Brevinin-1GHa and its two analogues were chemically synthesized by solid phase peptide synthesis and the second structures were predicted by online analysis tools. Subsequently, the synthetic peptides were subjected to antimicrobial and hemolysis assay to determinate their bioactive functions. On account of the strong antimicrobial activity of Brevinin-1GHa, more tests were carried, including time-killing curves, cell membrane permeability assay, and anti-biofilm assay.

## 2. Results

### 2.1. ‘Shotgun’ Cloning of a Novel Peptide Precursor-Encoding cDNA and Bioinformatic Analyses

**Figure 1:**
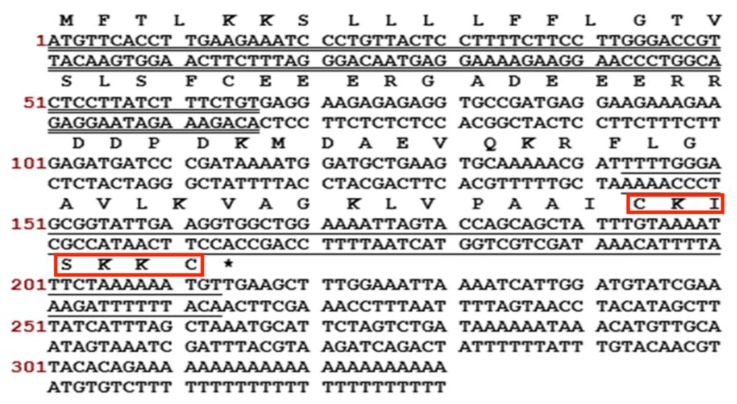
Nucleotide and translated open-reading frame amino acid sequence of a cDNA encoding the biosynthetic precursor of a novel Brevinin-1GHa from *H. guentheri*. The signal peptide is double-underlined, the mature peptide is single-underlined, and the stop codon is indicated by an asterisk. The Rana-box was indicated in the red boxes.

A full-length cDNA, encoding Brevinin-1GHa, was successfully cloned from the skin secretion –derived cDNA library of *H. guentheri*. The sequence of nucleotide and translated open reading frame amino acids are shown in Figure 1. There were several typical structural characteristics, including a putative signal peptide region of 22 amino acid residues, an acidic spacer peptide region, a classical -KR- propeptide convertase processing site, a mature peptide of 24 amino acid residues and a Rana-box at the C-terminal. The nucleotide sequence of Brevinin-1GHa precursor was deposited in the Genbank Nucleotide Sequence Database under the accession number MH791156.

The putative mature peptide was subjected to bioinformatics analysis by use of the National Center for Biotechnology Information (NCBI) protein Basic Local Alignment Search Tool (BLAST) program, which found that Brevinin-1GHa was a new Brevinin-1. The alignment demonstrated that Brevinin-1GHa owned at least eight different amino acid residues from others and showed 67% degree of structural identity to other Brevinin-1 peptides. Besides, the predicted secondary structures indicated Brevinin-1GHa had the highest proportion of α-helix among its homologs, as shown in Table 1.

### 2.2. Fractionation of Skin Secretion, Identification, and Structural Characterisation of Brevinin-1GHa

**Figure 2:**
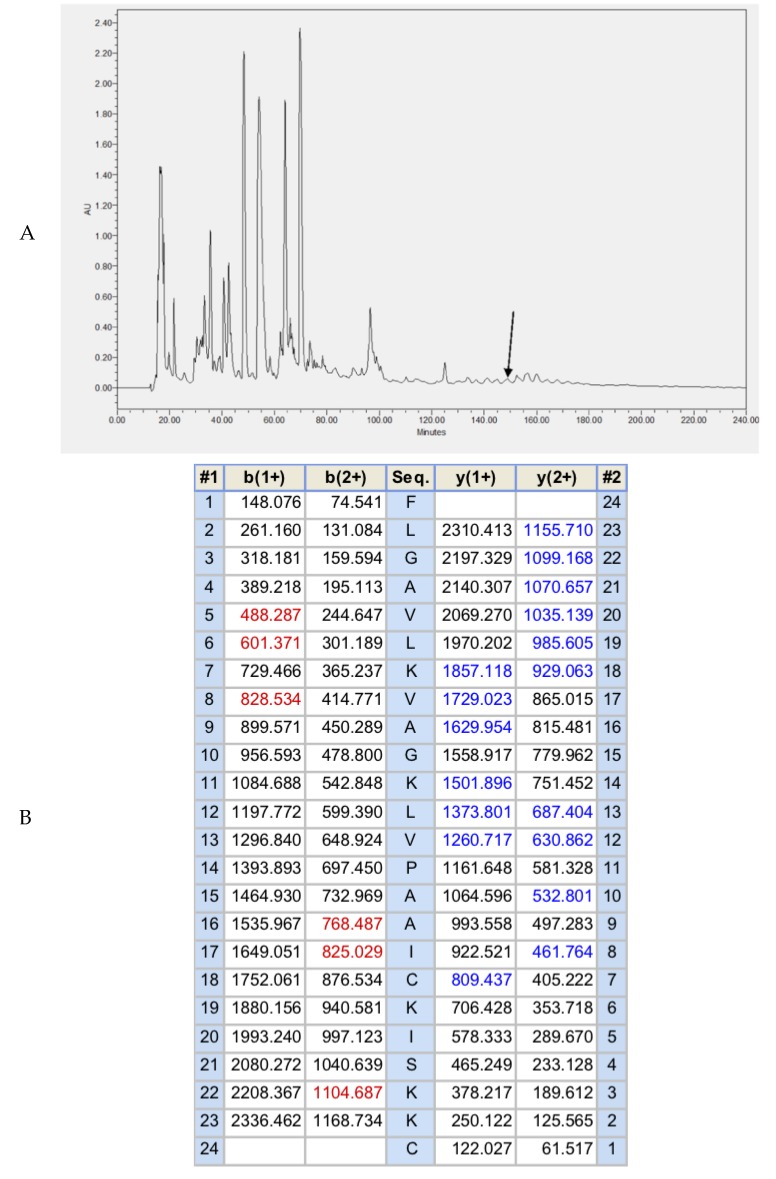
(**A**) RP-HPLC of skin secretion of *Hylarana guentheri* at a wavelength of 214 nm. The abscissa was ‘retention time (min)’, and the ordinate ‘AU’ was ‘absorbance’. The arrow indicated the retention time of Brevinin-1GHa. (**B**) Predicted b-ion and y-ion MS/MS fragment ion series (singly- and doubly-charged) of Brevinin-1GHa. The actual ions detected in MS/MS spectra are colored red and blue. #1, the sequence from N-terminal to C-terminal; b(1+) and b(2+), singly- and doubly-charged b-ions; Seq.,the sequence of amino acid residues. y(1+) and y(2+), singly- and doubly-charged y-ions; #2, the sequence from C-terminal to N-terminal.

The lyophilized crude skin secretion of *H. guentheri* was fractioned by reversed-phase high-performance liquid chromatography (RP-HPLC), as shown in Figure 2A. The arrow indicated the retention times/elution position of the peptide with the mass coincident with the approximate predicted molecular mass of Brevinin-1GHa. The amino acid sequence of Brevinin-1GHa, as further determined by MS/MS sequencing, is shown in Figure 2B.

### 2.3. Solid Phase Peptide Synthesis (SPPS) of Brevinin-1GHa, Brevinin-1GHb, and Brevinin-1GHc

After the determination of the primary structure of Brevinin-1GHa (FLGAVLKVAGKLVP AAICKISKKC), the peptide was successfully synthesized by applying a solid phase Fmoc chemical method. As some research about Brevinin-1 family peptides found, the C-terminal cyclic heptapeptide domain can be replaced by a C-terminally-amidated residue, it suggested the Rana-box may not be crucial for the antimicrobial activity of peptides [15]. To detect whether “Rana-box” plays an important role in the antimicrobial function of Brevinin-1GHa or not, we designed and synthesized a novel peptide Brevinin-1GHb (FLGAVLKVAGKLVPAAI). In addition, the research about Brevinin-1E suggested transferring the C-terminal Rana-box to central position led to a considerable reduction of Brevinin-1E’s hemolytic activity without loss of antibacterial activity [16], so Brevinin-1GHc (FLGAVLKVCKISKKCAGKLVPAAI) was designed and synthesized. All synthetic peptides were subjected to RP-HPLC to determine the degree of purity and MALDI-TOF to establish the authenticity of the structure.

### 2.4. Secondary Structure Prediction of Brevinin-1GHa, Brevinin-1GHb, and Brevinin-1GHc

**Figure 3:**
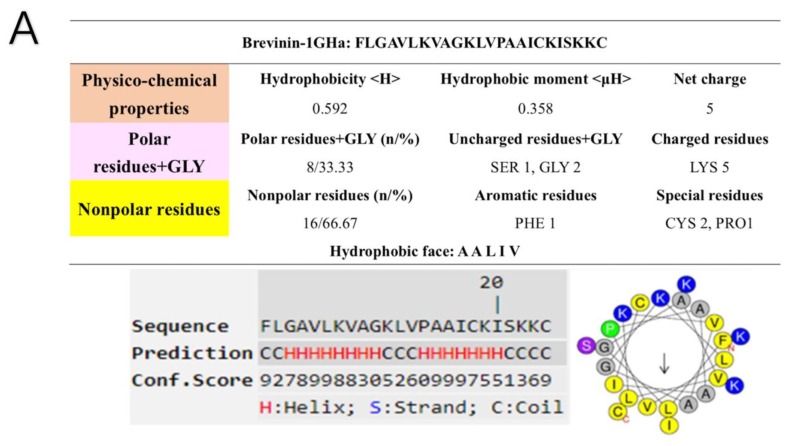

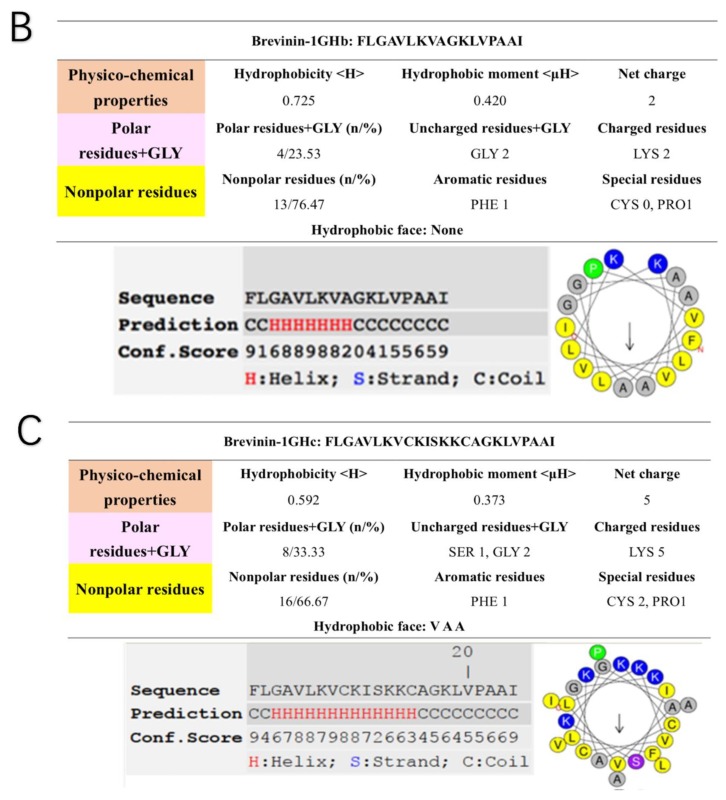
Structural parameters of peptides were predicted by the online analysis tool, HeliQuest, and I-TASSER. (**A**) Brevinin-1GHa; (**B**) Brevinin-1GHb; (**C**) Brevinin-1GHc.

The helical wheel plots and secondary structures were predicted by online analysis tools. The result showed that Brevinin-1GHa carried +5 net charge with the hydrophobicity of 0.592 and the hydrophobic moment of 0.358. Brevinin-1GHa also had a hydrophobic face consisted of A,A,L,I,V. Moreover, the predicted secondary structure contained a large proportion of the α-helical domain with a high C-score. Similarly, a three-dimensional simulation of Brevinin-1GHa exhibited the structural characteristics of α-helix, as shown in Figure 3A. Brevinin-1GHb only possessed two positive charges, with the hydrophobicity of 0.725 and the hydrophobic moment of 0.420. Different from Brevinin-1GHa, Brevinin-1GHb did not contain a hydrophobic face, and the length of α-helix is much shorter as shown in Figure 3B. Brevinin-1GHc owned five positive net charges, with the hydrophobicity of 0.592 and the hydrophobic moment of 0.373. Compared with Brevinin-1GHa, Brevinin-1GHc had a shorter hydrophobic face consisted of V,A,A, as shown in Figure 3C.

### 2.5. Antimicrobial and Hemolytic Activities of Brevinin-1GHa, Brevinin-1GHb, and Brevinin-1GHc

**Figure 4:**
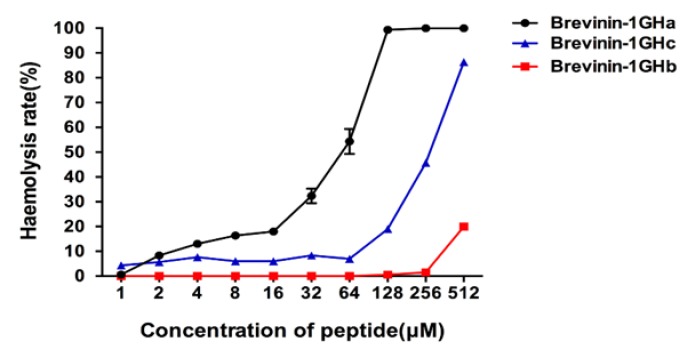
Hemolytic activity of Brevinin-1GHa, Brevinin-1GHb, and Brevinin-1GHc on horse erythrocytes.

To detect the antimicrobial activities of three synthesized peptides, we used S. aureus; Enterococcus faecalis (E. faecalis); methicillin-resistant Staphylococcus aureus (MRSA); E. coli; Pseudomonas aeruginosa (P. aeruginosa); Klebsiella pneumoniae (K. pneumoniae) and the yeast C. albicans. The results showed Brevinin-1GHa exhibited a potent broad-spectrum antimicrobial activity against all seven tested microorganisms. Brevinin-1GHb only showed the inhibition on S. aureus with the concentration at 512 µM. Brevinin-1GHc also inhibited the growth of seven microorganisms, but the efficacy is much lower than Brevinin-1GHa. The minimum inhibitory concentrations (MICs) and minimum bactericidal concentrations (MBCs) obtained with three peptides are shown in Table 2. Brevinin-1GHa, Brevinin-1GHb, and Brevinin-1GHc exhibited a hemolysis rate near 20% on horse erythrocytes with the concentration at 16, 512, and 128 µM, respectively, as shown in Figure 4.

### 2.6. Time-Killing Assay of Brevinin-1GHa against S. aureus, E. coli, and C. albicans

**Figure 5:**
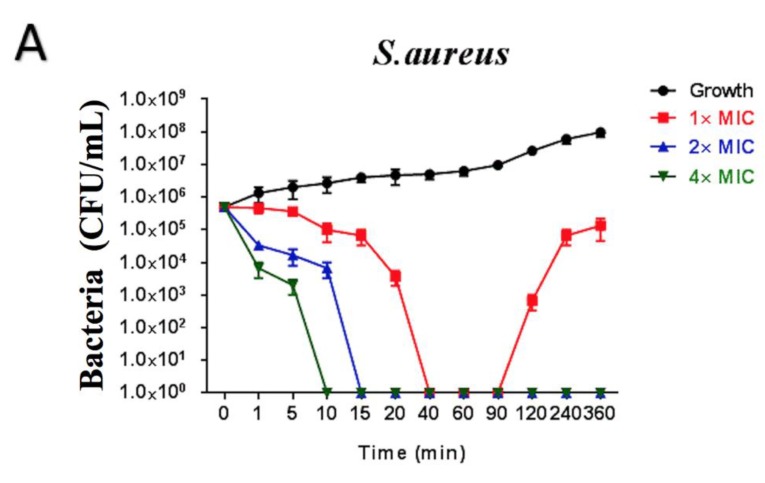

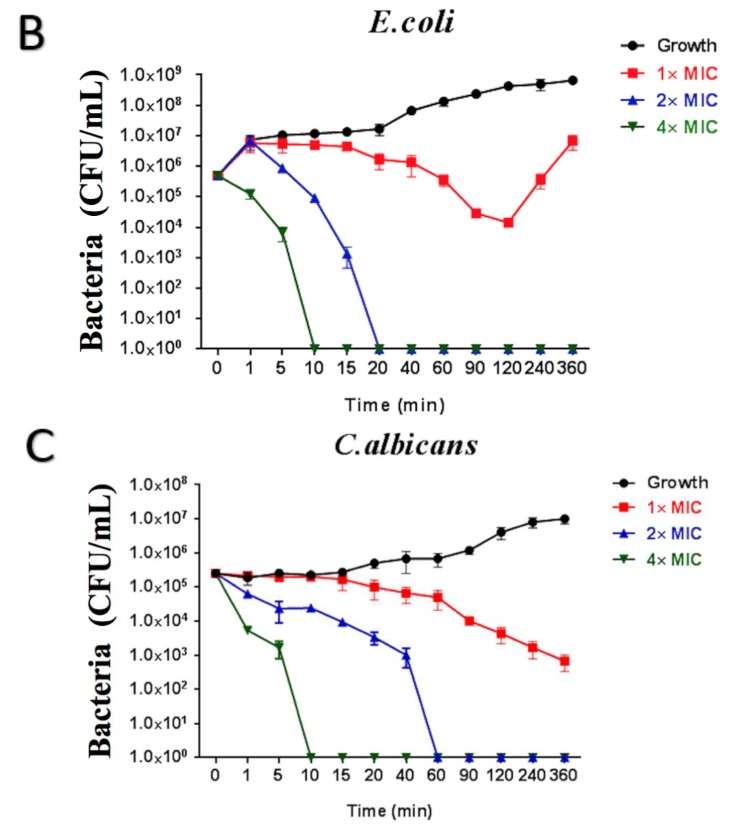
The time-killing curves of Brevinin-1GHa were tested with three concentrations at 1, 2, and 4× MIC. (**A**) *S. aureus*; (**B**) *E. coli*; (**C**) *C. albicans.*

Brevinin-1GHa with the concentration at 1× MIC inhibited the growth of *S. aureus* markedly during 20–40 min. Surviving cells could not be counted after 40 min, while it showed a recovery rate after 90 min. 2 and 4× MIC of Brevinin-1GHa killed bacterial to an uncountable state at 15 and 10 min, respectively, as shown in Figure 5A. *E. coli* was killed steadily by Brevinin-1GHa with concentration at 1× MIC during 360 min, and no bacterial was counted after 20 min treated with 2× MIC of Brevinin-1GHa. 4× MIC of Brevinin-1GHa inhibited the growth to an uncountable state at 10 min, as shown in Figure 5B. Similarly, *C. albicans* was reduced steadily by 1× MIC of Brevinin-1GHa. Treated with 2 and 4× MIC of Brevinin-1GHa, no yeast was counted at 60 and 10 min respectively, as shown in Figure 5C.

### 2.7. Cell-Membrane Permeabilization Assay of Brevinin-1GHa against S. aureus, E. coli, and C. albicans

**Figure 6:**
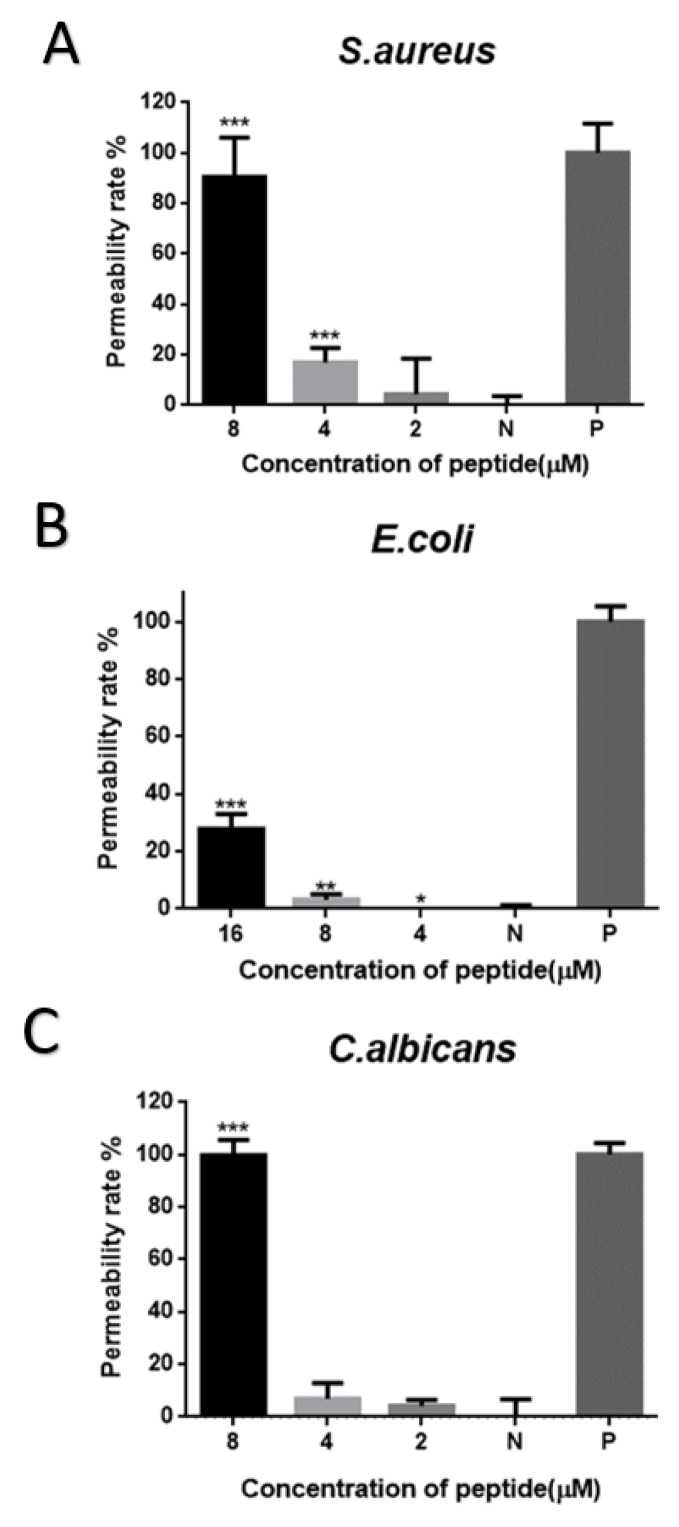
Cell-membrane permeability effects of Brevinin-1GHa on *S. aureus* (**A**); *E. coli* (**B**); *C. albicans* (**C**). Peptide concentrations corresponded to 1×, 2×, and 4× MIC. The positive controls were treated with 70% isopropanol, and the negative controls were represented as the vehicle only (*t*-test of each individual concentration contrast with negative control group, * *p* < 0.05, ** *p* < 0.01, *** *p* < 0.001).

Brevinin-1GHa did not cause the significant cell-membrane permeabilization of *S. aureus* with the concentration at 2 µM (1× MIC). While it showed the permeability rate at 20% and 90% with the concentration at 4 µM (2× MIC) and 8 µM (4× MIC), respectively, as shown in Figure 6A. Brevinin-1GHa only caused near 30% permeability rate of *E. coli* with the concentration at 16 µM (4× MIC), as shown in Figure 6B. Also, 8 µM (4× MIC) of Brevinin-1GHa disrupted the cell membrane of *C. albicans*, as shown in Figure 6C.

### 2.8. Anti-Biofilm Assay of Brevinin-1GHa against S. aureus, E. coli, and C. albicans

Brevinin-1GHa was able to suppress the biofilm formation of *S. aureus*, *E. coli,* and *C. albicans* broadly with the minimum biofilm inhibitory concentration (MBIC) at 4, 32, and 2 µM, respectively. Brevinin-1GHa could also eliminate the already formed biofilms with the minimal biofilm eradication concentration (MBEC) at 16 µM against *S. aureus*, 64 µM against *E. coli* and 8 µM against *C. albicans*, as shown in Table 3.

## 3. Discussion

Amphibians are the first group that bridges the evolutionary water−land gap and forced to face the dangerous environment including pathogenic microorganisms, predators, parasites, and physical factors. To adapt to the changing environment, amphibian skin which directly exposes to the outside world endowed with excellent chemical defense strategies. Lots of studies have proved that the skin secretion of amphibian can provide a significant amount of valuable information about prospective bioactive components, such as peptides, proteins, and alkaloids [17].

In this study, we cloned and purified a novel antimicrobial peptide from the skin secretion of *H. guentheri*. The amino acid sequence of Brevinin-1GHa was submitted to the BLAST program for the comparison of homology. The result revealed that the primary structure of Brevinin-1GHa is 67% identical to Brevinin-1HSa from *Odorranahosii*, Brevinin-1WY7 and Brevinin-1WY5 from *Amolopswuyiensis*, as well as Brevinin-1JDc from *Odorranajingdongensis*, as shown in Table 1. According to the UNIPORT database, only Brevinin-1HSa and Brevinin-1JDc’s bioactive functions have been detected; the results are shown in Table 4. Both Brevinin-1GHa and Brevinin-1JDc have demonstrated potent antimicrobial activities against the Gram-positive, Gram-negative and fungi tested, which may due to the strongly conserved structure formed by the Phe^1^, Ala^9^, Val^13^, and Pro^14^ residues [18]. The bacterial cell membrane is composed mainly of negatively charged phosphatidylglycerol and lipopolysaccharides. Therefore, the residues carried with the positive net charge in peptides are considered to play a significant role in binding to the phospholipid head groups in the cell membrane [19]. Then AMPs form hundreds of transient transmembrane pores that cause the leakage of small molecular mass molecules to facilitate the collapse of the cell membrane [20]. The 14th amino acid residue Proline of peptides in Brevinin-1 family produces a stable kink structure, which lets peptides be more likely to fasten on the zwitterionic membrane and facilitates the lysis of bacterial cell [21]. It explains why Proline is invariant but still exists in most of the Brevinin-1 peptides. Besides Pro^14^, the antimicrobial activities of AMPs against bacterial and fungi are determined by a complicated interaction between the grand average of hydropathicity (GRAVY), the theoretical isoelectric point (pI), cationicity, α-helix, hydrophobicity, and amphipathicity [22]. Although Brevinin-1GHa and Brevinin-1JDc show similar antimicrobial spectrum against Gram-positive and Gram-negative bacteria and fungi, Brevinin-1GHa inhibited the growth of both *S. aureus* (MIC = 2 μM), *E. coli* (MIC = 4 μM), and *C. albicans* (MIC = 2 μM) more effectively than Brevinin-1JDc. The difference may result from the increase in cationicity (Brevinin-1GHa pI = 9.90; Brevinin-1JDc pI = 9.85), which boosts the interaction with the cell membrane with a negative net charge and hence the antimicrobial activity of Brevinin-1GHa. With the highest net charge at +5, Brevinin-1GHa also owns an extremely high α-helix proportion at 83.33%; the amphipathic α-helix can destroy plasma membranes rapidly upon peptides associated with lipid cell membranes. In the end, hydrophobicity is an essential effector of antimicrobial activities as well. Brevinin-1GHa has the lowest hydrophobicity among peptides listed in Table 4, while just in the optimal hydrophobicity window could strong antimicrobial activity be obtained, higher hydrophobicity not only causes decreased antimicrobial activity, but also high hemolytic activity [23,24]. The reasonable explanation is strong peptide self-association occurs with high peptide hydrophobicity. It prevents peptides from passing through the cell membranes of prokaryotic cells but does not influence on eukaryotic cell walls [23].

Rana-box was thought to play a crucial role for antibacterial activities of peptides in Brevinin-1 family until the discovery of C-terminal truncated Brevinin-1 family peptide from *Rana okinavana*. The linear acetamidomethylcysteinyl analogue retaining its antibacterial activities indicates the disulfide bridge is not necessary for high potency [15]. Recent research has reported Brevinin-1RTa, and Brevinin-1RTb with Rana-box have weak inhibitory effects against Gram-negative bacteria [29]. To test whether Rana-box is significant for the antimicrobial activities of Brevinin-1GHa, Brevinin-1GHb was synthesized by SPPS without the disulfide bridge. Compared with Brevinin-1GHa, Brevinin-1GHb has a lower net charge at +2, a higher hydrophobicity at 0.725, and shorter length of the α-helix structure as the cut of Rana-box. All changes lead to the loss of antimicrobial activity, whereas the decrease in hemolytic activity. The decline in the inhibition of bacteria and fungi growth proves that Rana-box which provided the positive net charge plays a necessary role in the antimicrobial activities of Brevinin-1GHa. Contrast with higher hydrophobicity causing high hemolytic activity, Brevinin-1GHb without a hydrophobic face does not show hemolytic activity at a concentration of 256 μM. It indicates the hemolytic activity of peptides is not only related to the hydrophobicity, but also the hydrophobic face. As reported, transferring the Rana-box from C-terminal to central position leads to a considerable reduction of Brevinin-1E’s hemolytic activity without loss of antibacterial activity [14,16], so Brevinin-1GHc was designed and synthesized. The results revealed that the hemolytic activity increased dramatically at the concentration with 128 μM which is much lower than Brevinin-1GHa, while the antimicrobial activity was declined as well. The differences of physicochemical parameters between Brevinin-1GHa and Brevinin-1GHc are the hydrophobic moment (Brevinin-1GHa = 0.358; Brevinin-1GHc = 0.373) and the length of α-helix (Brevinin-1GHa = 83.33%; Brevinin-1GHc = 54.17%). It suggested the reduction of hemolytic activity and antimicrobial activity may mainly due to the decrease of α-helix length.

There are many antibacterial mechanisms, and the most widely accepted one is based on non-specific interaction with the membrane with the models, like barrel-stave mode, carpet mode, and toroidal mode [20,30,31]. According to the results of time-killing curves and the cell membrane permeability assay, the mechanism may be different between low and high concentration, which agreed with a previous publication [32]. With the low concentrations (1× MIC and 2× MIC) against *S. aureus* and *C. albicans*, Brevinin-1GHa may only cover a limited range of membrane leading to the formation of limited numbers or sizes of pores, while it nearly fully permeabilized cell membranes with the high concentration at 4× MIC. In addition, the membrane of *E. coli* was not destroyed, even with 4× MIC. The reasonable explanation is Gram-negative bacteria have an extra outer cell membrane with a large amount of highly negatively-charged lipopolysaccharide, so it becomes more difficult for AMPs to penetrate [33,34]. Biofilms, containing a polysaccharide matrix, are communities of microorganisms attached to a surface [35]. Biofilm-grown cells express several different properties, such as increased resistance to antimicrobial agents [36]. In the present study, Brevinin-1GHa exhibited the potent eradication and inhibition on the growth of biofilm against *S. aureus*, *E. coli*, and *C. albicans.*

In summary, a novel peptide Brevinin-1GHa was first isolated from the skin secretion of *H. guentheri*. Brevinin-1GHa showed a potent and broad spectrum of antimicrobial activities against Gram-positive bacteria, Gram-negative bacteria, and fungi. It was able to permeabilize the cell membrane of *S. aureus* and *C. albicans* with high concentrations and eradicate biofilms. However, with the aim of developing Brevinin-1GHa as a new potential antimicrobial drug, the modification should still be taken into consideration to reduce its hemolytic activity. This study suggests that Brevinin-1GHa is a promising candidate for the development of new antibiotic drugs and these findings may provide novel insights into natural antimicrobial drug design.

## 4. Materials and Methods

### 4.1. Acquisition of Skin Secretions

Specimens of the frog *H. guentheri* (*n* = 3) were captured from Fujian Province, People’s Republic of China. The skin secretions were obtained by mild transdermal electrical stimulation and mild massage on the skin of frogs, which then washed with deionized water. The combined viscous washings were dissolved in 0.05% (*v/v*) trifluoroacetic acid (TFA), snap-frozen in liquid nitrogen, lyophilized and stored at −20 °C [6]. The study was carried out according to the guidelines in the UK Animal (Scientific Procedures) Act 1986, project license PPL 2694, issued by the Department of Health, Social Services and Public Safety, Northern Ireland. Procedures had been vetted by the IACUC (PPL 2694) of Queen’s University Belfast, and approved on 1 March 2011.

### 4.2. Molecular Cloning of an *H. guentheri* Skin Secretion-Derived cDNA Library

Six milligrams of lyophilized skin secretion were dissolved in 1 mL of cell lysis/binding buffer (Life technologies, Oslo, Norway). Then according to the manufacturer, the polyadenylated mRNA was isolated with magnetic oligo-dT beads (Dynal, Merseyside, UK). A SMART-RACE kit (Clontech, Palo Alto, CA, USA) was utilized to achieve full-length prepropeptide nucleic acid sequence data. The 3′-RACE reaction employed a nested universal primer (NUP), and a degenerate sense primer (5′-TAYGARATHGAYAAYMGICC-3′ Y = C + T, R = A + G, H = A + T + C, M = A + C) was also designed to a conserved segment of the 5′-untranslated region. The PCR cycling program ran under the following condition: 90 s at 94 °C for initial denaturation; 35 cycles, 30 s at 94 °C for further denaturation; 30 s at 61 °C for primer annealing and 180 s at 72 °C for the extension. PCR products were analyzed by DNA-gel electrophoresis, purified and cloned by a pGEM-T vector system (Promega Corporation, Southampton, UK). A BigDye Terminator sequencing kit (Applied Biosystems, Foster City, CA, USA) was applied for sequencing reactions and the result was analyzed by an automated ABI 3100 DNA sequence.

### 4.3. Identification and Structural Analysis of Brevinin-1GHa

Five mg of lyophilized skin secretion of *Hylarana guentheri* was dissolved in 0.5 mL of trifluoroacetic acid (TFA)/water and centrifuged at 2500× *g* for 5 min. Then the supernatant was pumped to reverse-phase HPLC with the analytical Jupiter C-5 column (250 × 4.6 mm, Phenomenex, UK) and eluted with a 0–100% linear gradient program from water/TFA (99.95/0.05, *v/v*) to acetonitrile/water/TFA (80/19.95/0.05, *v/v/v*). The whole program ran over 240 min at a flow rate of 1 mL/min. Fractions were collected at 1 min intervals, and all samples were subjected to time-of-flight mass spectrometry (MALDI-TOF MS) (Voyager DE, Perspective Biosystems, Foster City, CA, USA). The instrument was calibrated in the range of 1–4 kDa, and the accuracy of mass determinations was ±0.1%. The fraction which contained peptides with same molecular mass as that predicted from cloned cDNA, was injected to analyses the primary structure by LCQ-Fleet ion-trap mass spectrometer (Thermo Fisher Scientific, San Francisco, CA, USA).

### 4.4. SPPS of the Novel Peptide and Its Analogues

Upon the primary structure of the novel peptide was determined, it was synthesized by solid-phase Fmoc chemistry using a Tribute automated peptide synthesizer (Protein Technologies, Tucson, AZ, USA). The amino acid sequence of the novel peptide was FLGAVLKVAGKLVPAAICKISKKC, and it was named Brevinin-1GHa. The amino acid sequences of two analogues were FLGAVLKVAGKLVPAAI and FLGAVLKVCKISKKCAGKLVPAAI, which were named as Brevinin-1GHb and Brevinin-1GHc respectively. All amino acids were weighed and mixed with the activator 2-(1H-benzotriazol-1-yl)-1,1,3,3,-tetramethyluronium hexafluorophosphate (HBTU). Dimethyl-formamide (DMF), N-methylmorpholine (NMM), and piperidine were also applied in the coupling program. Then the side chain protection groups were cleaved by the mixture composed of 94% Trifluoroacetic acid (TFA), 2% 1,2-ethanedithiol (EDT), 2% thioanisole (TIS), and 2% H_2_O at room temperature for 2 h with stirring. After cleavage, peptides were washed by diethyl ether and dissolved in 10 mL of acetonitrile/water/TFA (80/19.95/0.05, *v/v/v*) for lyophilization. The synthetic peptides were purified and confirmed by RP-HPLC and MALDI-TOF. Also, secondary structures of peptides were predicted by several online analysis tools, including HeliQuest and I-TASSER.

### 4.5. Antimicrobial Assay

Seven microorganisms were involved to detect the antimicrobial activity of three peptides, including the Gram-positive bacteria *Staphylococcus aureus* (*S. aureus*) (NCTC 10788), *Enterococcus faecalis* (*E. faecalis*) (NCTC 12697), methicillin-resistant *Staphylococcus aureus* (MRSA) (NCTC 12493); the Gram-negative bacterial *Escherichia coli* (*E. coli*) (NCTC 10418), *Pseudomonas aeruginosa* (*P. aeruginosa*) (ATCC 27853), *Klebsiella pneumoniae* (*K. pneumoniae*) (ATCC 43816) and the yeast *Candida albicans* (*C. albicans*) (NCYC 1467). All microorganisms were cultured in Mueller Hinton Broth (MHB) at 37 °C overnight and diluted to 5 × 10^5^ CFU/mL before adding to 96-well plates. Peptides were dissolved in DMSO to achieve the stock with the concentration at 51.2 mM and subsequently double-diluted to get the concentration of the peptide from 25.6 mM to 0.1 mM. 1 µL of peptide solutions were incubated with 99 µL cultured bacterial for 18 h at 37 °C. The minimal inhibitory concentrations (MICs) were determined at 550 nm by a Synergy HT plate reader (BioliseBioTek, Winooski, VT, USA). From wells which had no apparent growth of the microorganisms, 10 µL of solutions were dropped on Mueller Hinton agar (MHA) plates. MHA plates were cultured at 37 °C for 20 h, and the lowest concentrations with no colonies were the minimal bactericidal concentrations (MBCs).

### 4.6. Hemolytic Assay

A 2% (*v/v*) suspension of erythrocytes was prepared from prewashed defibrinated horse blood (TCS Biosciences Ltd., Buckingham, UK). Different concentrations of peptides from 512 µM to 1 µM were incubated with erythrocytes at 37 °C for 2 h. PBS in equal volumes was added as negative controls and 2% of the non-ionic detergent, Triton X-100 (Sigma-Aldrich, St. Louis, MO, USA), was added as positive controls.

### 4.7. Time-Killing Assay

*S. aureus*, *E. coli*, and *C. albicans* were cultured overnight in MHB medium to detect the time-killing curve. Three concentrations used of peptide Brevinin-1GHa were 4×, 2×, and 1× MIC, the MIC value was according to the results of antimicrobial assays. Bacterial and fungi were diluted with peptide-treated MHB to a concentration of 5 × 10^5^ CFU/mL. Growing bacterial and fungi were removed at specified time points and diluted to calculate the colonies.

### 4.8. Cell Membrane Permeability Assay

The membrane permeability assay was carried out by SYTOX Green Nucleic Acid Stain (Life technologies, Carlsbad, CA, USA). *S. aureus*, *E. coli*, and *C. albicans* were incubated in MHB at 37 °C overnight. *S. aureus* and *C. albicans* were subcultured in TSB, while *E. coli* was subcultured in LB. All of them were incubated at 37 °C for 2.5 h to achieve the logarithmic growth phase. Then cells were centrifuged and re-suspended in 5% TSB in 0.85% NaCl solution. 40 µL of peptide solutions with the concentration at 4×, 2×, and 1× MIC were incubated with 50 µL of cells suspension (1 × 10^7^ CFU/mL) for 2 h at 37 °C. Cells treated with 70% isopropanol were applied as positive controls, while 5% TSB were added as the negative control. After incubation, 10 µL SYTOX green was added into each well to achieve the final concentration at 5 µM. Plates were covered with tinfoil and incubated for 10 min. The fluorescent intensity was recorded by an ELISA plate reader (BioliseBioTek EL808) with excitation at 485 nm and emission at 528 nm.

### 4.9. Anti-Biofilm Assay

The anti-biofilm activities of Brevinin-1GHa were tested on *S. aureus*, *E. coli,* and *C. albicans*. All microorganisms were incubated in MHB overnight at 37 °C and subcultured, while *S. aureus*, *E. coli*, and *C. albicans* were diluted and cultured in tryptic soy broth (TSB), luria broth (LB), and 1640 medium respectively. For measuring the minimum biofilm inhibitory concentration (MBIC), peptide solutions from 1–512 µM (1 µL) were incubated with 5 × 10^5^ CFU/mL of bacterial or fungi (99 µL) in flat-bottomed 96-wells plates for 24 h. For measuring the minimum biofilm eradication concentration (MBEC), 100 µL of bacterial and fungi were added to the plates without peptides and incubated for 48 h. Until the biofilm formed, plates were washed by PBS and incubated with peptide solutions from 1–512 µM for 24 h. After incubation, all wells were washed by PBS and fixed by methanol for 30 min at room temperature. 0.1% Crystal Violet was used for staining and, 30% glacial acetic was used to dissolve the crystal after drying. The absorbance of each well was measured at 595 nm using the Synergy HT plate reader.

### 4.10. Statistical Analysis

Data were subjected to statistical analysis using Prism (Version 6.0; GraphPad Software Inc., San Diego, CA, USA). Error bars in the graphs represent standard error of the mean (SEM) with experiments performed on more than three sets of replicates.

## Figures and Tables

**Table 1:** Primary and secondary structural comparison of Brevinin-1GHa with relevant antimicrobial peptides (AMPs) homologs

Peptide Name	Source	Amino Acid Sequence	Alpha Helix	Identities
Brevinin-1GHa	*Hylarana guentheri*	^1^FLGAVLKVAGKLVPAAICKISKKC^24^ chhhhhhhhhhhhhhhhhhhhccc	83.33%	
Brevinin-1HSa	*Odorrana hosii*	^1^FLPAVLRVAAKIVPTVFCAISKKC^24^ cchhhhhhhhhhccceeeeecccc	41.67%	67%
Brevinin-1WY7	*Amolops wuyiensis*	^47^FLGSILGLVGKVVPTLICKISKKC^70^ cchhhhhhhhhhchheeeeecccc	50.00%	67%
Brevinin-1WY5	*Amolops wuyiensis*	^48^FLGSLLGLVGKVVPTLICKISKKC^71^ chhhhhhhhcccecceeeeeecccc	29.17%	67%
Brevinin-1JDc	*Odorrana jingdongensis*	^1^FLPAVLRVAAKVVPTVFCLISKKC^24^ cchhhhhhhhhhccceeeeeeccc	41.67%	67%
Brevinin-1RTc	*Amolops ricketti*	^48^FLGSLLGLVGKIVPTLICKISKKC^71^ chhhhhhhhhcecheeeeeecccc	41.67%	67%
Brevinin-1LTd	*Sylvirana latouchii*	^48^FFGSVLKVAAKVLPAAICQIFKKC^71^ cchhhhhhhhhhhhhhhhhhhhcc	83.33%	67%

The secondary structures of peptides were predicted by the online HNN program (http://npsa-prabi.ibcp.fr/cgi-bin/npsa_automat.pl?page=/NPSA/npsa_hnn.html). ‘h’ indicates α-helix, ‘c’ indicates random coil and ‘e’ indicates extended strand.

**Table 2:** Minimum inhibitory concentrations (MICs) and minimum bactericidal concentrations (MBCs) of Brevinin-1GHa, Brevinin-1GHb, and Brevinin-1GHc against seven different microorganisms

Microorganisms		Brevinin-1GHa		Brevinin-1GHb		Brevinin-1GHc	
		MIC (µM)	MBC (µM)	MIC (µM)	MBC (µM)	MIC (µM)	MBC (µM)
Gram-positive	*S. aureus*	2	2	512	>512	32	32
	*E. faecalis*	8	16	>512	>512	128	128
	MRSA	4	4	>512	>512	128	128
Gram-negative	*E. coli*	4	8	>512	>512	16	32
	*P. aeruginosa*	32	64	>512	>512	128	512
	*K. pneumoniae*	8	16	>512	>512	256	512
yeast	*C. albicans*	2	4	>512	>512	128	128

**Table 3:** Anti-biofilm activity of Brevinin-1GHa against the biofilm of *S. aureus*, *E. coli*, and *C. albicans*

Microorganisms	*S. aureus*	*E. coli*	*C. albicans*
MBIC (µM)	4	32	2
MBEC (µM)	16	64	8

**Table 4:** Structural parameters and minimum inhibitory concentrations (MICs) of Brevinin-1GHa and relevant AMPs homologs against specified microorganisms

Peptide Name	GRAVY	pI	Net Charge	<H>	<𝛍H>	Antimicrobial Activity (MIC 𝛍M)			
						*S. aureus*	*E. coli*	*C. albicans*	MRSA
Brevinin-1GHa	1.054	9.90	+5	0.592	0.358	2	4	2	4
Brevinin-1HSa	1.262	9.85	+4	0.728	0.377	3	24	ND	ND
Brevinin-1JDc	1.333	9.85	+4	0.761	0.366	6	49	3	3
Brevinin-1WY7	1.183	9.70	+4	0.736	0.420	ND	ND	ND	ND

GRAVY, grand average of hydropathicity; pI, theoretical isoelectric point. GRAVY and pI were calculated by the online ProtParam tool (http:web.expasy.org/protparam) [25]. <H>, hydrophobicity; <μH>, hydrophobic moment; Net charge, <H> and <μH> were calculated by the online analysis tool, HeliQuest (http://heliquest.ipmc.cnrs.fr) [26]. ND, not detected. MICs came from publications [27,28].
